# Genetics of Inherited Arrhythmias in Children

**Published:** 2008-05-01

**Authors:** Maully J Shah

**Affiliations:** Director, Interventional Electrophysiology, The Cardiac Center, The Children's Hospital of Philadelphia, 34th and Civic Center Blvd, Philadelphia, PA 19104

**Keywords:** inherited arrhythmias, children

Over the past two decades, breakthroughs in basic science have revealed the genetic etiology for several inherited arrhythmias. Onset of arrhythmias often commences in childhood and adolescence. The aim of the article is to provide a succinct overview of the genetic background of diseases that may cause life threatening arrhythmias in children and provide a description of reported genotype-phenotype relationships. Inherited channelopathies, namely, those causing long QT syndrome, short QT syndrome, catecholamine sensitive ventricular polymorphic ventricular tachycardia and Brugada syndrome and two cardiomyopathies (hypertrophic and arrhythmogenic right ventricular dysplasia) associated with ventricular arrhythmias are discussed.

## Inherited Channelopathies

### The Long QT Syndrome

The Long QT syndrome (LQTS) is an inherited arrhythmogenic disease occurring in the structurally normal heart that may cause sudden death and that usually manifests in children and teen-agers [1]. The estimated prevalence of this disorder is between 1:2,500 and 1:1,500. Two major phenotypic variants have been originally described in the early sixties: one autosomal dominant (Romano Ward syndrome) and one rare autosomal recessive (Jervell and Lange-Nielsen syndrome) also presenting with sensorineural deafness [2].

The LQTS is caused by an abnormal cardiac excitability. As a result, the affected patients have prolonged repolarization (QT interval at the surface electrocardiogram), abnormal T wave morphology, and life threatening cardiac arrhythmias. The mean age of onset of symptoms (syncope or sudden death) is 12 years and earlier onset is usually associated with more severe form of the disease [3]. Cardiac events are often precipitated by physical or emotional stress even though in a smaller subset of individuals cardiac events occur at rest [4]. For this reason antiadrenergic intervention with beta blockers is the cornerstone of therapy in the LQTS. For patients unresponsive to this approach, ICD and/or cardiac sympathetic denervation were proposed. It has been recently demonstrated that the response to beta blocker therapy is significantly modulated by the genotype and, specifically, the protection afforded by this approach is only partial for LQT2 and LQT3 patients [5,6].

#### Genetic Basis of Long QT Syndrome

As of today 10 LQTS genes have been identified. The typical LQTS phenotype with or without deafness may be due to mutations in 5 different genes while two variants present QT interval prolongation in the context of a multi-organ disease (Andersen syndrome) or with peculiar electrocardiographic features (LQT4). The discovery of the genetic basis of LQTS started in the early nineties with the mapping of four LQTS loci on chromosomes 11, 3, 7 and 4. The genes for these loci have been subsequently identified as KCNQ1 (LQT1) KCNH2 (LQT2) and SCN5A (LQT3) [7-9]. More recently mutations in two additional genes on chromosome 21, KCNE1 (LQT5) and KCNE2 (LQT6) were reported. All the LQT1-3 and LQT5-6 genes encode for cardiac ion channels subunits. LQT4 is caused by mutations in the ANK2 genes that encodes an intracellular protein called Ankyrin B that is involved in ion channels anchoring to the cellular membrane [10].

*LQT1, LQT5, JLN1, JLN2:* KCNQ1 (causing LQT1 and JLN1) and KCNE1 (causing LQT5 and JLN2) encode respectively for the alpha (KvLQT1) and the beta (MinK) subunits of the potassium channel conducting the IKs current, the slow component of the delayed rectifier current (IK) the major repolarizing current during phase 3 of the cardiac action potential. In order to form a functional channel, KvLQT1 proteins form homotetramers and it must also co-assemble with mink subunits.

LQT1 is the most prevalent genetic form of LQTS accounting for approximately 50% of genotyped patients. Several different mutations have been reported and in vitro expression studies of mutated proteins suggested multiple biophysical consequences all of them ultimately inducing a loss of function. Homozygous or compound heterozygous mutations of KCNQ1 also cause Jervell and Lange-Nielsen form of LQTS (JLN1) [2]. KCNE1 (LQT5) mutations are rather infrequent accounting approximately of 2-3% of genotyped LQTS patients and they may cause both Romano-Ward (LQT5) and, if homozygous, Jervell and Lange-Nielsen (JLN2) [12,1].

From a clinical standpoint LQT1 patients are those presenting a more straightforward adrenergic trigger for cardiac events. LQT1 is also characterized by the lower penetrance and a more benign prognosis compared with LQT2 and LQT3 [13].

*LQT2, LQT6:* KCNH2 (LQT2) and KCNE2 (causing LQT6) gene encode respectively for the alpha (HERG) and the beta (MiRP) subunits of the potassium channel conducting the IKr current the rapid component of the cardiac delayed rectifier. The KCNH2 encoded protein, HERG, forms homotetramers in the plasmalemma in order to make up functional channels [8]. The role or MiRP protein to recapitulate a fully operational current has been postulated but questioned by other authors. LQT2 is the second most common variant of LQTS accounting for 35%-40% of mutations. Functional expression studies have demonstrated that KCNH2 mutations cause a reduction of IKr current, but, similarly to LQT1 mutations, this effect is realized through different biophysical mechanisms and also though trafficking abnormalities of the mutant proteins [10]. LQT2 is characterized by higher penetrance and severity than LQT1, especially for females [13]. Mutations in the KCNE2 gene cause the LQT6 variant of LQTS, which is a very uncommon variant of the disease (<1%) and the associated phenotypes are characterized by incomplete penetrance and very mild manifestations.

*SCN5A (LQT3):* SCN5A encodes for cardiac sodium channel conducting the sodium inward current (INa). At variance with KvLQT1 and HERG proteins mutation, a single SCN5A transcript forms a fully functional channel protein (called Nav1.5). The first reported SCN5A mutations were clustered in regions functionally associated with channel inactivation. Subsequently several allelic variants have been reported and functional expression studies showed that, at variance with LQT1 and LQT2-asociated mutations, LQT3 defects cause a gain of function with an increased INa [9]. The prevalence of LQT3 among LQTS patient is estimated to be 10-15%, it is probably the more malignant form of LQTS and the one in which beta-blockers are less effective [13].

*ANK2 (LQT4):* The phenotype of the LQT4 patients differs from the typical LQTS. Most of the affected individuals, besides, QT interval prolongation, also present with severe sinus bradycardia, paroxysmal atrial fibrillation (detected in >50% of the patients) and with polyphasic T waves [14]. ANK2 encodes for an intracellular protein (Ankyrin B) that regulates the proper intracellular localization of plasmalemmal ion channels (calcium channel, sodium channel, sodium/calcium exchanger), sarcoplasmic reticulum channels (ryanodine receptor, inositol triphosphate receptor), and other adhesion molecules. The low number of LQT4 patients genotyped so far prevents the definition of prevalence (which appears low) and phenotypic features of this variant of LQTS. Additional ANKB mutations have been reported in patients with different forms of cardiac rhythm disturbances, thus further supporting the important role of this protein in the pathogenesis of cardiac arrhythmias [10].

*KCNJ2 (LQT7- Andersen syndrome):* In 2001, this disease was successfully mapped to the locus 17q in a large family. A candidate gene screening was carried out in the critical region and a heterozygous missense mutation was identified in the KCNJ2 gene. Additional mutations were subsequently identified in 8 unrelated individuals, thus providing the proof that KCNJ2 is the cause of at least some of AS. KCNJ2 encodes an inwardly rectifier potassium channel, Kir2.1, the ionic channel conducting the IK1 current. It is highly expressed in the heart where it appears to act as a determinant factor of phase 4 repolarization and of resting membrane potential. Interestingly, since dysmorphic facial appearance constitutes a distinctive trait of AS, the data also strongly suggest that Kir2.1 plays a major role in developmental signaling. Severe, functional impairment of IK1 current has been observed for all AS mutations so far studied with a variable degree of dominant negative effect when co-expression with wild type subunit [10].

*CACNA1c (LQT8 - Timothy syndrome):* Molecular screening of several cardiac ion channel encoding genes allowed the identification of a missense mutation (G406R) in the voltage-gated calcium channel gene (CACNA1c) [15]. Interestingly the same mutation was present in all probands analyzed and in one case parental mosaicism was demonstrated. Functional expression revealed that the G406R mutation produced maintained inward Ca(2+) currents by causing nearly complete loss of voltage-dependent inactivation. In the heart, prolonged Ca(2+) current delays cardiomyocyte repolarization and increases risk of arrhythmia. In other tissues and during fetal development this mutation is likely to cause intracellular calcium overload, a well known mechanism of tissue damage.

*CAV3 (LQT9):* Mutations in CAV3 that encodes for caveolin 3 have been linked to a rare form of long QT syndrome designated as LQT9 which has been also been associated with sudden infant death syndrome (SIDS) [16].

*SCNB4 (LQT10):* Mutations in SCNB4 are responsible for another rare type of long QT syndrome designated as LQT10 [17].

#### Genotype-Phenotype Correlation in Long QT Syndrome

In the last few years several studies have outlined the distinguishing features of the three most common genetic variants of LQTS (LQT1, LQT2, LQT3), which account for approximately 97% of all genotyped patients. Gene-specific repolarization morphology and gene-specific triggers for cardiac events have been described. LQT1 patients usually develop symptoms during physical activity, conversely LQT3 have events at rest. Auditory stimuli and arousal are a relatively specific trigger for LQT2 patients while swimming is a predisposing setting for cardiac events in LQT1 patients [3]. Gene-specific differences of the natural history of LQTS have also been demonstrated and allow genotype-based risk stratification [13]. Indeed, QT interval duration, genotype and gender are significantly associated with the outcome with a QTc interval >500ms, and a LQT2 or LQT3 genotype determining the worst prognosis. Gender differentially modulates the outcome according to the underlying genetic defect: the LQT3 males and LQT2 females are the highest risk subgroups. LQT8 is a rare variant of LQTS characterized by marked QT interval prolongation (often presenting with 2:1 functional atrioventricular block and macroscopic T wave alternans), and cutaneus syndactyly at both hands and feet. Severe prognosis has been observed in all cases described so far [15]. Individuals with Timothy syndrome (TS) also had congenital heart disease, including patent ductus arteriosus, patent foramen ovale, ventricular septal defects, and tetralogy of Fallot. Some children had dysmorphic facial features, including flat nasal bridge, small upper jaw, low-set ears, or small or misplaced teeth. Episodic serum hypocalcemia was described in 4 individuals. Many of the surviving children showed developmental delays consistent with language, motor, and generalized cognitive impairment. The definition of Andersen syndrome (AS) was used for the first time in 1994 by Tawil et al to describe a clinical disorder consisting of three major features: potassium-sensitive periodic paralysis, ventricular arrhythmias and dysmorphic features (short stature, hypertelorism, broad nasal root, and defect of soft and hard palate). An additional peculiar feature of AS is the presence of a variable degree of QT interval prolongation and abnormal T wave morphology often presenting with very prominent U waves. Life threatening ventricular arrhythmias are probably rare in AS although sudden death has been reported.

### Short QT Syndrome

The first report of the so-called "short QT syndrome" (SQTS) was provided by Gussak et al. who described a familial distribution of a persistently short QT interval associated paroxysmal atrial fibrillation in one patient [18]. They also reported similar ECG changes in an unrelated case associated with sudden cardiac death. More recently, these observation have been refined by Gaita et al. who described two families with severe history of sudden cardiac death, QTc interval constantly below 290ms, ventricular premature beats and documented ventricular fibrillation [19].

SQTS is also characterized by the absence of structural heart disease, a remarkable familial history of sudden cardiac death and a typical, hyperkalemic-like T wave pattern (tall and peak T wave) with a very fast terminal phase of repolarization on the resting ECG. ICD implantation is currently the treatment of choice for symptomatic patients with short QT syndrome and a family history of sudden cardiac death. Quindine has been shown to normalize ventricular repolarization (at variance with other antiarrhythmic drugs including sotalol and amiodarone) in some patients but at present its role in preventing recurrences of cardiac events is not known. Therefore, at present this approach may not be considered an alternative to ICD.

#### Genetic background of short QT syndrome

The prevalence of the SQTS is currently unknown and less than 50 families have been reported. In the two families reported by Gaita et al., genetic investigation led to the identification of a coding defect in the KCNH2 gene [19]. In vitro functional characterization demonstrated a remarkable increase of IKr current, and reduced the affinity of the channels to IKr blockers [20]. Thus, it is suggested that, at variance with the LQT2 variant of Long QT Syndrome associated with loss of function KCNH2 mutation, the SQTS phenotype is caused by gain of function mutations. More recently, Bellocq et al. reported another sporadic case of SQTS with a missense mutation of the KCNQ1 gene. In vitro expression demonstrated a gain of function with an increase of the IKs current. In March 2005 Priori et al. identified a novel SQTS locus (SQTS3) by reporting a gain of function mutation in the KCNJ2 gene, encoding the inward rectifier potassium channel which is a voltage-gated channel responsible for the IK1 current [21].

Thus, SQTS is a genetically heterogeneous disease. Genotype-phenotype correlation and clinical data on natural history, risk stratification and optimal therapeutic management are still lacking.

### Catecholaminergic polymorphic ventricular tachycardia

Catecholaminergic polymorphic ventricular tachycardia (CPVT) is a familial arrhythmogenic disorder characterized by adrenergically mediated polymorphic ventricular tachyarrhythmias [22]. It is an important cause of syncope and sudden cardiac death in individuals with a structurally normal heart and usually a normal QT interval on the surface ECG. If left untreated, 80% of patients will develop symptoms by the age of 40 years and the overall mortality is 30-50% [23].

#### Genetic background of catecholaminergic polymorphic ventricular tachycardia

Recently, genetic investigations have identified two variants of the disease: an autosomal dominant form associated with mutations in the gene encoding the cardiac ryanodine receptor (RyR2) and a recessive form associated with homozygous mutations in the gene encoding the cardiac isoform of calsequestrin (CASQ2). The first advancement in the understanding of the pathophysiology of CPVT was made possible by the linkage studies that were able to localize the CPVT locus to the long arm of chromosome 1 (1q42-q43). Subsequently, Priori and coworkers using the candidate gene approach screened the open reading frame of the RyR2 gene encoding the cardiac RyR2 and identified pathogenic mutations in four CPVT probands [24]. RyR2 is a protein functionally involved in the Ca^2+^ release from the sarcoplasmic reticulum in response to the activation of the ICa^2+^ current through the dihydropyridine receptor during the plateau phase of the action potential. The clear adrenergic-mediated onset of arrhythmias closely resembles the arrhythmias developing during intracellular calcium overload and delayed afterdepolarization. A few years later, advancement in the study of CPVT was obtained when the autosomal recessive variant of CPVT was linked to chromosome 1 (1p13-p21) and established that the disease gene is encoding for cardiac calsequestrin (CASQ2), another protein strictly implicated in the control of intracellular calcium [25].

Taken together the experimental data demonstrated that both genes involved in CPVT pathogenesis affect the amount of Ca2+ released from the SR during adrenergic stimulation. Such effect may create an electrically unstable substrate probably through triggered activity-mediated arrhythmogenesis.

### Brugada Syndrome

Brugada syndrome (BrS) is an inherited arrhythmogenic disease characterized by ST segment elevation in the right precordial leads (V1 to V3), right bundle branch block (either complete and incomplete) and susceptibility to ventricular tachyarrhythmia, typically during rest or sleep [264]. Supraventricular tachyarrhythmias like atrial fibrillation are also a common finding in these patients.

Brugada syndrome is inherited as autosomal dominant trait, but the majority of the individuals developing ventricular arrhythmias are of male gender (male to female ratio: 8:1). Furthermore, despite the genetic defect is present since birth the disease tends to manifest in the third decade of life. Indeed, syncope or cardiac arrests occur more often in the third to fourth decade of life. Only rare instances of malignant forms with earlier onset during childhood or neonatal manifestations have been reported. The causes of age- and gender- related penetrance of BS are unclear.

#### Genetic background of Brugada syndrome

Mutations in the cardiac sodium channel gene, SCN5A, on chromosome 3p21-23, have been identified for the first time in 1998. This gene encodes for the alpha subunit of the cardiac sodium channel protein (identified as Nav1,5), which is the most important player in determining the phase 0 of the cardiac action potential (depolarization), thus it controls a crucial step in the physiology of electrical activation of the heart [27]. Interestingly, BrS is not the only phenotype linked to SCN5A mutations. Known allelic disorders are: the LQT3 variant of Long QT syndrome, the progressive cardiac conduction defect and the sick sinus syndrome [28]. Furthermore, recent findings also suggest that SCN5A mutations may be present in patients with typical BrS electrocardiogram but also myocardial abnormalities resembling those typical for arrhythmogenic right ventricular cardiomyopathy (ARVC) and in families with dilated cardiomyopathy (DCM) [10]. SCN5A has a 6.048 Kb coding sequence spanning and approximately 65% of mutations so far reported in this gene are associated with a BrS phenotype but no specific clustering within the coding region is demonstrable. Interestingly, not only ORF mutations but also genetic variants in the SCN5A promoter may have pathophysiologic role in BrS. A haplotype block of six SNP in the SCN5A promoter has been identified and functionally linked to a reduced expression of the sodium current. This variant was found among subjects of Asian origin (allelic frequency 0.22) and it could play a role in modulating the expression of BrS in far east countries where the disease is thought to be particularly frequent. In vitro expression of mutant SCN5A proteins showed that BrS mutations invariably cause a loss of sodium channel function at variance with the LQT3 mutation that cause an excess of sodium current. The most frequent biophysical mechanisms are reduced current density, slower recovery of inactivation, shift of voltage dependence of inactivation and an enhancement in the intermediate inactivation. Mutations in the SCN5A gene may also modify the interaction with the sodium channel beta-subunit and with other proteins controlling intracellular localization of the channel. The second, recently identified BrS gene is called GPD1-L, encoding for the Glycerol-3-Phosphate-Dehydrogenase 1-like protein [29]. A mutation was reported to co-segregate with the BrS phenotype in a single large family. The function of GPD1-L is poorly known at present, but preliminary in vitro data suggest that it controls the membrane expression of the sodium channels and that the mutation reduces the amount of sodium current similar to that observed for other BrS mutations affecting SCN5A. Quinidine, a non-specific blocker of cardiac transient outward current (Ito) has been proposed as gene-specific therapy for BrS. This proposal is based the idea that by blocking repolarizing currents, and specifically Ito, which is active during the initial phases of action potential, quinidine could restore the equilibrium between inward (constitutively reduced in BrS) and outward currents. The available clinical data show that quinidine prevents arrhythmia inducibility at PES and suggest a positive long-term effect in preventing the occurrence of spontaneous arrhythmias. Although no final proof of effectiveness is available quinidine may be regarded as an adjunctive therapy for patients at higher risk and to reduce the number of ICD shock in patients with multiple recurrences.

## Cardiomyopathies associated with ventricular arrhythmias

### Hypertrophic Cardiomyopathy

Hypertrophic cardiomyopathy (HCM) is the most prevalent genetic cardiovascular disease affecting one in 500 individuals, and is the most common cause of sudden cardiac death in young athletes [30]. It is remarkable for its genetic and phenotypic heterogeneity and can manifest with negligible to extreme hypertrophy, minimal to extensive fibrosis and myocyte disarray, absent to severe left-ventricular outflow tract obstruction, and distinct septal contours/morphologies such as reverse curve, sigmoidal, and apical variant-HCM. The clinical course also varies extremely, ranging from an asymptomatic lifelong course to dyspnea/angina refractory to pharmacotherapy to sudden death as the sentinel event.

#### Genetic background of hypertrophic cardiomyopathy

Since the sentinel discovery of the first locus for familial HCM (1989) and the first mutations involving the MYH7-encoded beta myosin heavy chain (1990) as the pathogenic basis for HCM, over 300 mutations scattered among at least 24 genes encoding various sarcomeric, calcium-handling and mitochondrial proteins have been identified [31,32]. The most common genetically mediated form of HCM is myofilament HCM, with hundreds of disease-associated mutations in eight genes encoding proteins critical to the sarcomere's thick myofilament [beta-myosin heavy chain (MYH7), regulatory myosin light chain (MYL2) and essential myosin light chain (MYL3)], intermediate myofilament (myosin binding protein C; MYBPC3), and thin myofilament [cardiac troponin T (TNNT2), alpha-tropomyosin (TPM1), cardiac troponin I (TNNI3), and actin (ACTC)] [33]. More recently, mutations have been described in the myofilament protein alpha-myosin heavy chain encoded by MYH6 [33]. In the past, it was thought that specific mutations in these myofilament genes were inherently 'benign' or 'malignant', genotype-phenotype studies involving a large cohort of unrelated patients have indicated that great caution must be exercised in assigning particular prognostic significance to any particular mutation. Furthermore, those studies have demonstrated that the two most common forms of genetically mediated HCM, MYH7 HCM and MYBPC3 HCM, are phenotypically indistinguishable [34]. The prevalence of mutations in the eight most common myofilament-associated genes, currently comprising the commercially available HCM genetic test (www.hpcgg.org) in different international cohorts, ranges from 30 to 61%, still leaving a large number of patients with genetically unexplained disease.

Over the past few years, the spectrum of HCM-associated genes expanded outside the myofilament to encompass additional subgroups that could be classified as 'Z-disc HCM', 'calcium-handling HCM', and 'metabolic HCM'. As a result of its close proximity to the contractile apparatus of the myofilament and its specific structure-function relationship with regards to cyto-architecture, as well as its role in the stretch-sensor mechanism of the sarcomere, recent attention has been focused on the cardiac Z-disc. Initial mutations were described in muscle LIM protein encoded by CSRP3 and telethonin encoded by TCAP [35]. LDB3-encoded LIM domain binding 3, ACTN2-encoded alpha actinin 2 and VCL-encoded vinculin/metavinculin have recently been added to that list. Interestingly, although the first HCM-associated mutation in vinculin was found in the cardiac-specific insert of the gene, yielding the protein called metavinculin, the follow-up study also identified a mutation in the ubiquitously expressed protein vinculin.

As the critical ion in the excitation-contraction coupling of the cardiomyocyte, calcium and proteins involved in calcium-induced calcium release have always been of great interest in the pathogenesis of HCM. Although with very low frequency, mutations have been described in the promoter and coding-region of PLN-encoded phospholamban, an important inhibitor of cardiac muscle sarcoplasmic reticulum Ca2+-ATPase (SERCA) as well as in the RyR2-encoded cardiac ryanodine receptor [36]. Three novel mutations in JPH2-encoded junctophilin 2 in three, previously genotype-negative, patients with HCM have been recently discovered. This is the first time that JPH2, which is thought to play a role in approximating the sarcoplasmic reticulum calcium release channels and plasmalemmal L-type calcium channels, has been implicated in the pathogenesis of HCM [33].

The last important genetic subgroup of HCM is that of metabolic HCM, involving mitochondrial and lysosomal proteins. In 2005, Arad and colleagues first described mutations in lysosome-associated membrane protein 2 encoded by LAMP2 and protein kinase gamma-2 encoded by PRKAG2 in glycogen storage disease-associated genes mimicking the clinical phenotype of HCM [37]. In 2005, a mutation in FXN-encoded frataxin was described in a patient with HCM. Although this patient also harbored a myofilament mutation in MYBPC3-encoded myosin binding protein C, functional characterization showed significant influence of the FXN mutant on the phenotype, suggesting that the observed alterations in energetics may act in synergy with the present myofilament mutation. Similar to PRKAG2 and LAMP2, Fabry's disease can express predominant cardiac features of left ventricular hypertrophy. Over the years, mutations in GLA-encoded alpha-galactosidase A have been found in patients with this multisystem disorder [33].

Although up to 24 HCM susceptibility genes involving different pathways have been identified, the search for novel mutations in new genes continues. A genome-wide linkage study recently identified a new locus for HCM in a large family with left ventricular hypertrophy located on chromosome 7. Subsequent studies of genes located in this region, however, have so far not yielded the causative gene [33].

#### Genotype-phenotype correlations in hypertrophic cardiomyopathy

Several studies have tried to identify phenotypic characteristics of myofilament/sarcomeric HCM to facilitate genetic counseling and strategically direct clinical genetic testing. Although several phenotype-genotype relationships have emerged to increase the yield of genetic testing, these patient profiles have not been particularly clinically informative. Echocardiographically determined septal morphology was recently linked to the underlying genetic substrate in which the septal contour, classified as reverse septal contour, sigmoidal septal contour, apical, and neutral contour. In the early 1990s Solomon et al. described an early genotype-phenotype observation involving a small number of patients and family members, and discovered that patients with mutations in the beta myosin heavy chain (MYH7 HCM) generally had reversed curvature septal contours (reverse curve HCM).

Recently, a large genotype-phenotype analysis correlating the septal morphology with the underlying genotype. In an analysis of the echocardiograms of 382 previously genotyped and published patients, it was observed that sigmoidal HCM (47% of the cohort) and reverse curve HCM (35% of the cohort) were the two most prevalent anatomical subtypes of HCM, and discovered that the septal contour was the strongest predictor of the presence of a myofilament mutation, regardless of age. The yield of the commercially available HCM genetic test for myofilament HCM was 79% in reverse curve HCM but only 8% in patients with sigmoidal HCM. Of the smaller subgroup of patients with apical HCM, 32% had a mutation in one of the myofilaments. These observations may facilitate echo-guided genetic testing by enabling informed genetic counseling about the a-priori probability of a positive genetic test based upon the patient's expressed anatomical phenotype. In addition, the paucity of myofilament mutations in sigmoidal HCM opens the door for research to elucidate the molecular/genetic determinants of sigmoidal HCM [39].

### Arrhythmogenic right ventricular dysplasia or cardiomyopathy

Arrhythmogenic right ventricular dysplasia or cardiomyopathy (ARVD) is a heart muscle disease, often familial, that is characterized pathologically by atrophy and fibro-fatty replacement of the right ventricular myocardium and clinically by ventricular arrhythmias of right ventricular origin or sudden death [39,40]. The progressive loss of the RV myocardium has been related to a genetically determined dystrophic process, an inflammatory myocardial injury and death, or a programmed cell death ("apoptosis").

The clinical presentation of ARVD peculiarly consists of arrhythmias of right ventricular origin ranging from isolated premature ventricular beats to sustained ventricular tachycardia or ventricular fibrillation leading to sudden death. Other clinical manifestations of the disease include global and/or regional dysfunction and structural alterations of the right ventricle, ECG depolarization/repolarization changes characteristically in right precodial leads, and evolution to right or biventricular heart failure mimicking dilated cardiomyopathy [41].

#### Genetic background of Arrhythmogenic right ventricular dysplasia/cardiomyopathy

Several loci of ARVD have been mapped. In addition to ARVD1 on 14q23-q24, these include ARVD2 on 1q42-q43, ARVD3 on 14q12-q22, ARVD 4 on 2q32.1-q32.3, ARVD5 on 3p23, ARVD6 on 10p13-p12, ARVD7 on 10q22.3, ARVD 8 on 6p24, ARVD9 on 12p11, ARVD10 on 18q12.1-q12.2, and ARVD 11 on 18q21 [42]. The causative gene in ARVD1 is TGFB3, ARVD2 is RYR2, in ARVD5 is LAMR1, in ARVD8 DSP and in ARVD9 PKP2, in ARVD10DSG2, and in ARVD11 DSC2. ARVD2 is allelic with CPVT. Tiso et al suggested that ARVD2 associated mutations increase RYR2 mediated calcium release to the cytoplasm where as CPVT associated mutations do not significantly affect cytosolic calcium levels. ARVD7 is a type of Desmin related myopathy in which there are intrasarocplasmic aggregates of Desmin. Myopathy may be associated with cardiomyopathy. ARVD8 is caused by mutation in the gene encoding desmoplakin (DSP). Gerull et al speculated that lack of plakophilin-2 or incorporation of mutant plakophilin in the cardiac desmosomes in ARVD9 impairs cell to cell contacts and as a consequence disrupts adjacent cardiomyocytes in response to stretch and stress. Intercellulardisruption would occur first in areas of high stress and stretch: RV outflow tract, apex and sub-tricuspid area (triangle of dysplasia) [41]. The potential for ventricular arrhythmias in ARVD is the intrinsic variation in conduction properties as a result of patchy areas of fibrofatty myocyte degeneration. The desmosomal cadherins are potential cell adhesions molecules of the desmosome type of cell junction. Two classes have been identified: desmogleins and desmocollins. Mutations in DSG2 have been correlated with ARVD10 and DSC2 with ARVD11 [43].

## Conclusion

Despite recent advances in the understanding of the genetic background of inherited arrhythmias, genetic testing is still in its infancy. In families with inherited arrhythmias, if a pathogenic mutation is identified by genetic testing, it becomes possible to establish a pre-symptomatic diagnosis of the disease among other family members. In certain instances, where predictable genotype-phenotype correlations have been established, the results can provide important information to drive management or assist in reproductive counseling. As the genetic basis of arrhythmias continues to unravel, a transition from reactive and suppressive management to a new paradigm of rational and pre-emptive treatment to prevent the expression of genetic mutations is envisioned.
